# Efficacy of biphasic cuirass ventilation in the critical care department

**DOI:** 10.1186/cc10739

**Published:** 2012-03-20

**Authors:** T Yamashita, Y Taniwaki, H Takayama, Y Sakamoto

**Affiliations:** 1 Saga University, Saga, Japan; 2 National Hospital Organization Nagasaki Medical Center, Omura, Japan

## Introduction

Biphasic cuirass ventilation (BCV) assists ventilation by applying intermittent or continuous negative pressure to the thorax. BCV has been reported to improve lung function in various respiratory failures. However, to determine the therapeutic effect of BCV is difficult, because it is too difficult to include animal experiments. Therefore it is important to compile amounts of clinical cases for discussion. We have tried to find a way of developing BCV in critical care.

## Methods

This is a retrospective, nonrandomized study. Before and after BCV, we compared pO_2_, pCO_2_, tidal volume, P/F ratio, respiratory index, A-aDO_2_, shunt ratio, dead space ventilation rate, and chest X-ray. We also performed a questionnaire study about BCV which focused on physicians and nurses working in the ICU.

## Results

From April 2008 to May 2010, BCV was performed by applying RTX (Medivent Ltd, London, UK) for 18 patients admitted to the ICU, National Hospital Organization Nagasaki Medical Center. All of them had acute respiratory failure, and 15 of them were intubated and mechanically ventilated. Thirteen were men, and the mean age was 68 years (1 to 82 years). One case could not continue the treatment due to discomfort of wearing the cuirass. We used the control mode (negative pressure -21 cmH_2_O, positive pressure +7 cmH_2_O, I:E ratio 1:1). It improved the tidal volume, P/F ratio, shunt ratio in all cases during BCV (*P *< 0.05). Skin damage caused by the cuirass was observed in one case. According to the questionnaire survey, they had some problems about the durability of the urethane of the cuirass, too close to a thin body or deformation. Some of them had no confidence because of unfamiliarity with the machine.

## Conclusion

We conclude that BCV is also useful for respiratory care in the ICU. Further confirmation is needed regarding problems such as the criteria to start and terminate BCV.
